# Phase I trial outcome of amnion cell therapy in patients with ischemic stroke (I-ACT)

**DOI:** 10.3389/fnins.2023.1153231

**Published:** 2023-05-09

**Authors:** Thanh G. Phan, Rebecca Lim, Siow T. Chan, Hannah McDonald, Poh-Yi Gan, Shenpeng R. Zhang, Liz J. Barreto Arce, Jason Vuong, Tharani Thirugnanachandran, Benjamin Clissold, John Ly, Shaloo Singhal, Marie Veronic Hervet, Hyun Ah Kim, Grant R. Drummond, Euan M. Wallace, Henry Ma, Christopher G. Sobey

**Affiliations:** ^1^Clinical Trials, Imaging and Informatics Division, Stroke and Ageing Research, Department of Medicine, School of Clinical Sciences at Monash Health, Monash University, Clayton, VIC, Australia; ^2^Department of Neurology, Monash Health, Clayton, VIC, Australia; ^3^The Ritchie Centre, Hudson Institute of Medical Research, Clayton, VIC, Australia; ^4^Department of Obstetrics and Gynaecology, Monash University, Clayton, VIC, Australia; ^5^Department of Medicine, Centre for Inflammatory Diseases, Monash Medical Centre, School of Clinical Sciences, Monash University, Clayton, VIC, Australia; ^6^Department of Immunology, Monash Health, Monash Medical Centre, Clayton, VIC, Australia; ^7^Department of Microbiology, Anatomy, Physiology and Pharmacology, Centre for Cardiovascular Biology and Disease Research, School of Agriculture, Biomedicine and Environment, La Trobe University, Bundoora, VIC, Australia; ^8^Victorian Department of Health, Melbourne, VIC, Australia

**Keywords:** stem cell, clinical trial, ischemic stroke, phase I, allogeneic

## Abstract

**Background:**

We proposed a Phase I dose escalation trial to assess the safety of allogeneic human amniotic epithelial cells (hAECs) in stroke patients with a view to informing the design for a Phase II trial.

**Methods:**

The design is based on 3 + 3 dose escalation design with additional components for measuring MR signal of efficacy as well as the effect of hAECs (2–8 × 10^6^/kg, i.v.) on preventing immunosuppression after stroke.

**Results:**

Eight patients (six males) were recruited within 24 h of ischemic stroke onset and were infused with hAECs. We were able to increase the dose of hAECs to 8 × 10^6^ cells/kg (2 × 10^6^/kg, *n* = 3; 4 × 10^6^/kg, *n* = 3; 8 × 10^6^/kg, *n* = 2). The mean age is 68.0 ± 10.9 (mean ± SD). The frequencies of hypertension and hyperlipidemia were 87.5%, diabetes was 37.5%, atrial fibrillation was 50%, ischemic heart disease was 37.5% and ever-smoker was 25%. Overall, baseline NIHSS was 7.5 ± 3.1, 7.8 ± 7.2 at 24 h, and 4.9 ± 5.4 at 1 week (*n* = 8). The modified Rankin scale at 90 days was 2.1 ± 1.2. Supplemental oxygen was given in five patients during hAEC infusion. Using pre-defined criteria, two serious adverse events occurred. One patient developed recurrent stroke and another developed pulmonary embolism whilst in rehabilitation. For the last four patients, infusion of hAECs was split across separate infusions on subsequent days to reduce the risk for fluid overload.

**Conclusion:**

Our Phase I trial demonstrates that a maximal dose of 2 × 10^6^/kg hAECs given intravenously each day over 2 days (a total of 4 × 10^6^/kg) is safe and optimal for use in a Phase II trial.

**Clinical trial registration:**

ClinicalTrials.gov, identifier ACTRN12618000076279P.

## Introduction

Stroke is the second leading cause of death and third leading cause of disability and death worldwide and results in significant economic and societal cost (GBD 2019 Stroke Collaborators, 2021). The Global Burden of Disease reports that there were 12.2 million incident cases of stroke in 2019 (GBD 2019 Stroke Collaborators, 2021). There are currently reperfusion therapies, including the clot-busting drugs (alteplase and tenecteplase) and mechanical thrombectomy for large clots (Saver et al., 2016). The national registry from North America showed that in 2018 less than 19.1 and 9.1% of patients received thrombolysis and mechanical thrombectomy, respectively (Akbik et al., 2020). This type of treatment is effective in patients who have an identifiable ischemic penumbra or salvageable tissue on multimodality imaging studies. Among patients receiving thrombectomy, a substantial proportion (30.9%) attained independent status at discharge but 29.7% required assistance with walking, 24.0% were unable to walk and 15.4% died (Akbik et al., 2020). There is thus an urgent need for adjunctive therapies for stroke such as neuroprotective agents (Hill et al., 2020) and cell therapy (Hess et al., 2017).

There is interest in cell therapy as a novel treatment modality, especially for patients who are unable to receive reperfusion therapy or for whom these treatments have failed (Phan et al., 2018). Cell therapy is typically based on the premise that the cells attenuate injury mechanisms and enhance the recovery process. Human amniotic epithelial cells (hAECs) have been safely transplanted into humans without evidence of immune rejection for decades, and we have reviewed their suitability as an inherently safe and amenable stroke therapy (Broughton et al., 2013; Evans et al., 2018a). hAECs are obtained from readily available discarded term placentae, and so their use avoids invasive extraction procedures and significant ethical constraints. They are immunologically inert with low levels of human leukocyte antigens A, B, C, and DR—key antigens involved in transplant rejection—as well as expression and release of HLA-G, which is able to directly suppress immune responses. Thus, hAEC transplantation does not require adjunct immunosuppressant administration. Moreover, hAECs lack telomerase enzyme and so do not form tumors, nor do they differentiate into fibroblasts. Chemoattraction to sites of injury, where there are large amounts of SDF-1 released, occurs via the CXCR4 receptor expressed on the cell surface (Evans et al., 2018b).

Our prior studies have demonstrated the efficacy of hAECs in reducing infarct volume and improving functional outcome in different animal models of ischemic stroke, including non-human primates (Evans et al., 2018b). Indeed, hAEC therapy could be demonstrated to improve outcomes even when administered 3 days after stroke onset. The key mechanisms of action involved modulation of the harmful post-stroke inflammatory response to reduce secondary injury in the peri-infarct region. The aim of this study was to evaluate the safety and maximum tolerable dose of hAECs in patients in the acute phase of ischemic stroke (Phan et al., 2018). We also assessed infarct volume and FLAIR-DWI mismatch, hematological profile including lymphocyte subsets, and inflammatory markers over the first 7 days post-treatment. The trial is registered on the Australian New Zealand Clinical Trials Registry (ACTRN12618000076279p).

## Materials and methods

The clinical protocol for this Phase I trial was previously published (Phan et al., 2018). We have provided a summary of the inclusion and exclusion criteria below. The dosing schedule was calculated by lean body weight in two patients with large body habitus (Patients 6 and 8). Due to low recruitment rate, we modified the original plan of our Phase I trial to include patients who received thrombolysis. This step was supported by our animal experiments showing that hAECs provided neuroprotection when administered in combination with alteplase (manuscript in preparation). The trial was terminated with eight patients recruited when the primary endpoint of the trial (identification of a safe dose range and the maximum tolerated dose) was deemed to have been met.

### Inclusion criteria

Patients were eligible if: (1) they had ischemic stroke in the territory of the large main artery (middle cerebral artery) (Phan et al., 2009); (2) presented within 24 h of stroke onset and were not eligible for TPA or clot retrieval; (3) aged between 18 and 85 years old; and (4) had a National Institute of Health Stroke Scale/NIHSS score (tool used in clinical trials for measuring stroke severity) of between 6 and 15.

### Exclusion criteria

Patients were excluded if there was evidence of: (1) autoimmune disease, organ transplant, malignancy, splenectomized individuals, or had infection at the time of stroke; (2) neurodegenerative disease such as dementia or Parkinson’s disease; (3) pregnancy; (4) have contraindications for magnetic resonance (MR) imaging [patients with initial infarct (on DWI) volume <5 ml was excluded] (MR Stroke Collaborative Group et al., 2006); (5) mild stroke (NIHSS <6) or very severe stroke (NIHSS >15); and (6) eligible for TPA and/or ECR. During the course of the trial, we obtained an amendment from Monash Health Human Research Ethics Committee to include patients who had received TPA but less than 4 points reduction in NIHSS.

### Treatment protocol

Doses of hAECs administered intravenously have ranged from 1 × 10^6^/kg in human infants with bronchopulmonary dysplasia (Lim et al., 2018) to 30–40 × 10^6^/kg in mice with experimental stroke (Evans et al., 2018a). Here, doses chosen for stage-wise escalation were: 2 × 10^6^, 4 × 10^6^, 8 × 10^6^, 16 × 10^6^, and 32 × 10^6^ cells/kg. Patients were given hAECs by intravenous infusions over 1 h (Phan et al., 2018). At higher doses, patients were given hAECs over 2 or 3 divided doses (Table 2).

### Sample size

This trial was designed to identify a safe dose range, including the maximum tolerated dose (O’Quigley et al., 1990). The dose escalation and de-escalation stages were designed in keeping with the principles of the classic 3 + 3 scheme in a phase I trial whereby the sample size is increased in small increments of 3. For a 5-dose escalation, the estimated sample size is 15 (Liu and Yuan, 2015).

### Statistical analysis

Descriptive statistics were used to describe patient demographics, safety and SAE of hAECs. Regression analyses were not used given the eventual sample size of 8.

### Image analysis

Images were de-identified prior to analysis and performed by JV. Segmentation of infarct was performed using MRIcroGL software (freely available at https://www.nitrc.org/plugins/mwiki/index.php/mricrogl:MainPage).

### Immunological assay

We performed laboratory studies of blood samples to evaluate the effects of the hAECs on the immune response (highly sensitive C reactive protein, MMP-9, IL-6, IL-10, IL-17, IFN-gamma, CD3, CD4, and CD19) post-stroke using pre-defined methods.

### Data sharing

De-identified patient data will be available after 2 years. Requests for data can be made to the principal investigator. The study protocol is available at ANZCTR trial website and in previous publication (Phan et al., 2018).

## Results

Eight patients (six males) were recruited. Clinical characteristics and rationale for treatment are provided in Table 1. The mean age is 68.0 ± 10.9. The frequencies of hypertension and hyperlipidemia were 87.5%, diabetes was 37.5%, ischemic heart disease was 37.5%, atrial fibrillation was 50% and ever-smoker was 25%. The baseline NIHSS was 7.5 ± 3.1, at 24 h was 7.8 ± 7.2, and at 1 week was 4.9 ± 5.4. The modified Rankin scale at 90 days was 2.1 ± 1.2.

**TABLE 1:** Patient characteristics and outcome.

Patient	Dose (×10^6^ cells/kg)	Weight (kg)	Age	Arterial occlusion	Perfusion mismatch (%)	TPA	NIHSS day 0	NIHSS day 7	NIHSS day 30	mRS 90 days	mRS 1 year
1	2	90	60 s	M2	0	No	6	3	1	1	1
2	2	90	60 s	M2	50	No	6	1	1	1	1
3	2	86	70 s	M2	50	No	6	0	0	0	0
4	4	70	70 s	A2	100	No (Apixaban)	7	12	16	4	4
5	4	102	70 s	M2	50	No	6	mRS = 3*	mRS = 2*	0	n/a*
6	4	135^+^	40 s	M2	50	No	6	2	2	1	1
7	8	86	70 s	M2	25	No (Dabigatran)	15	10	10	4	4
8	8	119^+^	60 s	M2	25	No	8	7	4	3	1

*Patient 5: Unable to perform NIHSS on day 7 and day 30 due to COVID-19 restrictions. mRS was instead performed via Telehealth; Unable to contact patient for 1 year follow-up. ^+^Patient dose was calculated by lean body weight. TPA, tissue plasminogen activator, M2 is distal segment of middle cerebral artery, A2 is distal segment of anterior cerebral artery.

**TABLE 2:** Intravenous infusion of allogeneic hAEC in acute stroke patients.

Patient	Dose (×10^6^ cells/kg)	Weight (kg)	Total dose (×10^6^ cells)	Infusion 1 Days post-stroke	Infusion 1 dose (×10^6^ cells)	Infusion 2 Days post-stroke	Infusion 2 dose (×10^6^ cells)	Infusion 3 Days post-stroke	Infusion 3 dose (×10^6^ cells)
1	2	90	180	1	180	NA	NA	NA	NA
2	2	90	180	1	180	NA	NA	NA	NA
3	2	86	160	1	160	NA	NA	NA	NA
4	4	70	280	1	280	NA	NA	NA	NA
5	4	102	408	1	204	2	204	NA	NA
6	4	135*	330	1	115	2	115	NA	NA
7	8	86	688	1	230	2	230	3	228
8	8	119*	484	1	161	1^+^	161	4^+^	162

NA, not applicable. *Patient dose was calculated by lean body weight. ^+^Timing of the 2nd and 3rd infusions for Patient 8 were impacted by a shutdown of cell preparation facilities on Days 2 and 3 post-stroke.

### Imaging characteristics

**FIGURE 1:**
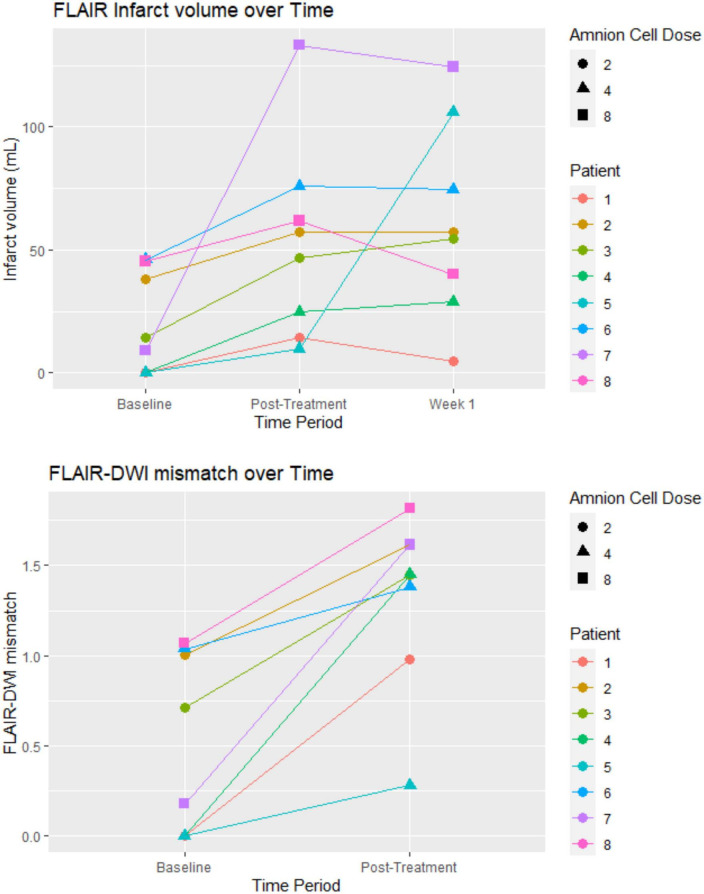
Infarct volume and FLAIR-DWI mismatch: the post-treatment was on the day that the hAEC infusion was completed. For patients receiving a dose of 8 × 10^6^ cells/kg the MRI was on day 2. The unit of hAEC dose was 10^6^ cells/kg. FLAIR, fluid attenuated inversion recovery sequence; DWI, diffusion weighted imaging.

Most of the patients in the study had more than 50% perfusion mismatch between cerebral blood volume and cerebral mean transit time. No patient was given alteplase due to the patient being outside of the time window or judged to have ischemic injury in at least 50% of the vascular territory. This decision was made by the treating clinicians. Another patient (patient 4) could not be given alteplase due to therapeutic apixaban level. Figure 1 illustrates the change in infarct volume over the first week. In seven of eight patients, there was no marked increase in infarct volume after cell infusion was completed. Patient 4 had recurrent stroke on the day after receiving hAECs and patient 7 had significant infarct growth prior to completion of hAEC infusions but without clinical deterioration in NIHSS. The NIHSS decreased from 15 on admission to 10 at 7 days (Table 1). Median infarct volume on FLAIR imaging at baseline was 11.74 ml (IQR 0, 39.89). Median ischemic volume on DWI at baseline was 29.10 ml (IQR 6.99, 42.92). Median infarct volume at post-treatment was 52.05 ml (UQR 22.2, 65.4). Median infarct volume at 1 week was 55.77 ml (IQR 37.26, 82.28).

### Hematological profile

**FIGURE 2:**
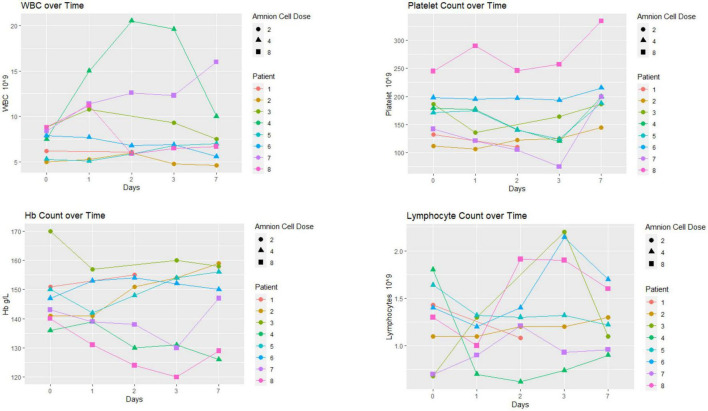
Hematological profile: there was no particular pattern observed among the eight patients. The unit of hAEC dose was ×10^6^ cells/kg.

Hematological changes are displayed in Figure 2. There was no particular pattern observed among the 8 patients. Four patients had increases in white blood cells (WBC) above 10 × 10^9^/L. The elevation in WBCs was transient in two of these four patients. The Chest XR, blood and urine cultures in these patients were normal. The hemoglobin profile did not dip below 120 g/L. The platelet count dipped below 100 × 10^9^/L in patient 7 on day 3 before returning to normal on day 7. The profile showed transient lymphopenia (below 1 × 10^9^/L) in patient 4. Eosinophil counts were 0.10 ± 0.10 × 10^9^/L (mean ± SD) at baseline and remained in the normal range at 5–7 days following cell infusion (0.15 ± 0.10 × 10^9^/L, *n* = 8; data not shown).

### Immunological profile

**FIGURE 3:**
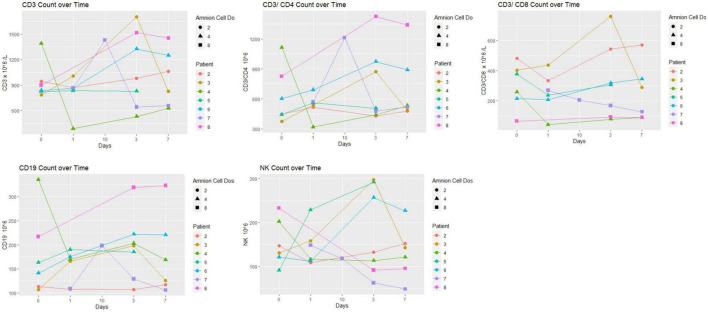
Lymphocyte subset over time: there was no particular pattern observed among the eight patients. The unit of hAEC dose was ×10^6^ cells/kg.

**FIGURE 4:**
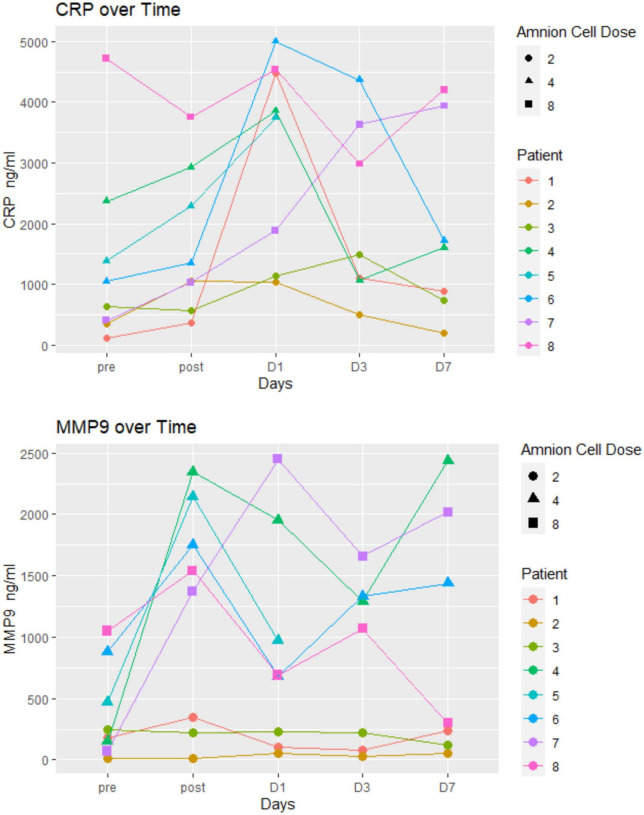
Inflammatory markers: there was no particular pattern observed among the eight patients. The unit of hAEC dose was ×10^6^ cells/kg.

Immunological changes are displayed in Figures 3, 4. There was no particular pattern observed among the 8 patients. In brief, the results show no obvious trends, especially in terms of effect of hAECs (e.g., between patients 7 and 8 that received the highest dose and patients 1–3 who received the lowest dose). We were not able to detect (or detected low levels) of several inflammatory markers (IFN-γ, IL-6, TNF, IL-10, and IL-17). Levels of high sensitivity CRP and MMP-9 remained within normal range. In three patients (patients 1–3), MMP-9 levels did not change over the first week.

### Safety

For the last four patients the infusion of hAECs was split across separate infusions on subsequent days to reduce risk for fluid overload (Table 2). This enabled us to increase the dose of hAECs to 8 × 10^6^ cells/kg. Supplemental oxygen was given in 5 patients during hAEC infusion (Table 3). These patients did not require oxygen therapy toward the end of the cell therapy procedure. Using pre-defined criteria, two serious adverse events occurred. One patient developed recurrent stroke 2 days after admission in the setting of atrial fibrillation (patient 4) and another patient developed pulmonary embolism 19 days later, whilst in rehabilitation (patient 7). No patient died within 1 year of trial enrolment. Overall, the data indicate that a dose of 2 × 10^6^/kg hAECs given intravenously each day over 2 days (a total of 4 × 10^6^/kg) is safe and optimal for use in a Phase II trial.

**TABLE 3:** Adverse and serious adverse effects.

Patient	Dose (×10^6^ cells/kg)	Age	AE1	AE1 Classification	AE1 Severity	AE2	AE2 Classification	AE2 Severity	AE3	AE3 Classification	AE3 Severity	SAE	SAE classification	SAE Severity
1	2	60 s	Supplemental Oxygen	Causal	Mild	NA	NA	NA	NA	NA	NA	No	NA	NA
2	2	60 s	Supplemental Oxygen	Causal	Mild	NA	NA	NA	NA	NA	NA	No	NA	NA
3	2	70 s	No	NA	NA	NA	NA	NA	NA	NA	NA	No	NA	NA
4*	4	70 s	Fluid overload	Causal	Moderate	Infection	Probable	Mild	Supplemental Oxygen	Mild	Mild	Recurrent Stroke	Probable	Severe
5	4	70 s	Supplemental Oxygen	Causal	Mild	NA	NA	NA	NA	NA	NA	No	NA	NA
6	4	40 s	No	NA	NA	NA	NA	NA	NA	NA	NA	No	NA	NA
7^+^	8	70 s	Pneumonia	Probable	Moderate	NA	NA	NA	NA	NA	NA	Pulmonary Embolism	Probable	Severe
8	8	60 s	Supplemental Oxygen	Causal	Mild	NA	NA	NA	NA	NA	NA	No	NA	NA

AE, Adverse effect; SAE, Serious adverse effect; NA, not applicable. *Patient 4 had recurrent stroke on day 2 of admission or 1 day after cell therapy. This had occurred in the setting of atrial fibrillation. The recurrence stroke rate within 30 days among patients with minor stroke and atrial fibrillation has been estimated at 6.3% (on dabigatran) to 9.8% (on aspirin) (Liu and Yuan, 2015). The patient also had fluid overload and was given treatment with diuretics. This was initially diagnosed as pneumonia as the patient had high white cell count and the initial chest x-ray report was consolidation. Six days later, the chest x-ray was reported as fluid overload. Due to the potential for fluid overload, we changed the infusion protocol and slow down the rate of infusion in Phase I. ^+^Patient 7 developed pulmonary embolism on day 19 while in rehabilitation. Pulmonary embolism is not unexpected outcome of patients with stroke. The prevalence of pulmonary embolism in the first 30 days was 0.78% among 11,287 patients (Pongmoragot et al., 2013). On day 1 of admission, the patient developed pneumonia. This was documented on chest x-ray and was treated with antibiotics. The frequency of pneumonia in our hospital is 6.6% and was more common among patients with moderate or severe stroke (Phan et al., 2019). This patient’s initial NIHSS was 15.

## Discussion

In this small Phase I dose escalation trial, we have shown safety of allogeneic hAEC in patients in the acute phase of ischemic stroke. With the exception of 2 patients, the majority of patients in this trial had minimal to mild disability at 3 months and at 1 year follow-up. As this was a dose escalation trial without a comparator arm, we were not able to determine the effects of hAECs. The lessons in this Phase I trial have been used to inform the design of a multisite Phase II trial where comparator arms will be included.

Intravenous administration of hAECs in the acute phase of ischemic stroke with the goal of modulating the inflammatory response in our study has similarity with a trial of multipotent adult progenitor cells (Hess et al., 2017). This approach is different from other stem cell therapies in the chronic phase of stroke which have used invasive stereotactic implantation of an immortalized human neural stem-cell line (Muir et al., 2020) or modified bone marrow-derived mesenchymal stem cells (Steinberg et al., 2016). The different therapeutic strategies reflect different intended modes of action of stem cells. Notably, hAECs are stem-like cells of epithelial origin.

Our approach is feasible within the first 24 h of stroke and had 2 SAEs. It is possible that the SAE of recurrent stroke within 2 days in the setting of atrial fibrillation was an expected complication of stroke rather than a reflection of hAEC infusion. The recurrence stroke rate within 30 days among patients with minor stroke and atrial fibrillation has been estimated at 6.3% (on dabigatran) to 9.8% (on aspirin) (Butcher et al., 2020). Similarly, pulmonary embolism is a known complication of stroke and its occurrence 19 days after onset was not unexpected. The prevalence of pulmonary embolism in the first 30 days was 0.78% among 11,287 patients and is higher among patients with more severe stroke deficit (Pongmoragot et al., 2013). While we cannot discount the possibility of pulmonary embolism being related to cell therapy, we expect that such an event would have occurred earlier in the course of stroke rather than at day 19 while the patient is attending rehabilitation. Furthermore, the baseline NIHSS for this patient was 19, which would have placed them at high risk of pulmonary embolism. Data from a large-scale Canadian study reported that the odds ratio was 4.37 for patients with moderately severe ischemic stroke and 12.93 for severe stroke (Pongmoragot et al., 2013).

The primary aim of this study was dose escalation and an embedded imaging component was included to obtain insight into infarct expansion/growth ratio as a surrogate marker to inform the design of a Phase II trial (MR Stroke Collaborative Group et al., 2006). The observed growth in the infarct lesion between baseline and first week in our study had also been described in other neuroprotection trials (Warach et al., 2000; Kidwell et al., 2009). In conjunction with the mismatch between clinical outcome and infarct growth it is likely that infarct growth ratio may not be the best surrogate marker in neuroprotection or stem cell trials (MR Stroke Collaborative Group et al., 2006). For example, infarct growth was observed in patient 7, yet the NIHSS trended in the opposite direction. Infarct growth as detected by MRI is not unexpected given the presence of a penumbra on the CT perfusion and a clot within a cerebral vessel. Indeed, infarct expansion ratio may be a better marker in thrombectomy trials (Tate et al., 2021).

In this Phase I trial we used the opportunity to evaluate the hematological and immunological profiles following hAECs. Our interest in the levels of various immune cells and cytokines was based on the knowledge that pro-inflammatory mechanisms can exacerbate post-stroke outcomes and that hAEC therapy might be expected to mitigate against this (Broughton et al., 2013). In general, levels of all markers remained in the normal range and in some cases were only transiently changed over the 7 days following stroke. As this Phase I trial did not include a placebo arm for comparison with the hAEC-treated group, we were not able to evaluate change in inflammatory response and the plots of the hematological and immunological tests did not show a consistent profile in relation to hAEC dose. The inflammatory markers (high sensitivity CRP and MMP-9) in our cohorts were not elevated and some (IFN-γ, IL-6, TNF, IL-10, and IL-17) were below the limits of detection. It is possible that a number of the samples may have degraded after storage for 2–3 years, as analyses were only performed after the trial was closed. Other investigators have described a linear relationship between MMP-9 and infarct volume (Rosell et al., 2005) as well as increased MMP-9 and fatal hemorrhagic transformation (Rosell et al., 2008). An elevated level of high sensitivity CRP, particularly to >7 mg/L, has been associated with poor outcome at 3 months (den Hertog et al., 2009). CRP levels in our patients were much lower, yet 2 of the patients had a high Rankin score at 3 months. The low CRP is consistent with difficulty in detecting the other inflammatory markers. Alternatively, low levels of inflammatory markers might indicate a modulating effect of hAECs on the systemic inflammatory response in this cell-treated cohort.

Five patients had transient hypoxemia during cell transfusion but did not require oxygen therapy toward the end of the cell infusion. Whether the requirement for oxygen therapy is due to cell trapping is not known as amnion cells are small relative to the size of the vessel and of mesenchymal stem cells (Broughton et al., 2013). We acknowledge that transient hypoxia could be a risk associated with infusion of cells, and the risk and severity of associated AEs/SAEs would increase as the dose of cells or density of cell suspension increases. However, it is difficult to adjudicate such a risk, as all efforts are made to avoid hypoxia during the infusion process. However, we have also observed this phenomenon in pre-clinical studies and that the risk of losing animals due to pulmonary embolism increases with the dose of cells infused.

## Conclusion

In this cohort, intravenous infusion of hAECs was found to be safe at a dose of up to 8 × 10^6^ cells/kg, but a dose of 2 × 10^6^/kg hAECs given intravenously each day over 2 days (a total of 4 × 10^6^/kg) was deemed to be optimal for use in a Phase II trial. This finding has been used to design the protocol for Phase II trial of Amnion Cell Therapy for Ischemic Stroke (ACT-2).

## Data availability statement

The original contributions presented in this study are included in the article/supplementary material, further inquiries can be directed to the corresponding authors.

## Ethics statement

The studies involving human participants were reviewed and approved by the Monash Health Human Research Ethics Committee. The patients/participants provided their written informed consent to participate in this study.

## Author contributions

TP, HMa, RL, EW, and CS designed the trial protocol and were major contributors in the writing of the manuscript. JV performed image analysis. TT, BC, JL, and SS recruited patients and performed clinical assessments. RL, SC, and HMa prepared cells for administration to patients. MH administered cell infusions and collected the patient data. P-YG, SZ, LB, and HK performed the analyses of blood samples for inflammatory markers. CS and GD analyzed the data. All authors contributed to the article and approved the submitted version.
