# Corrigendum: Phase I trial outcome of amnion cell therapy in patients with ischemic stroke (I-ACT)

**DOI:** 10.3389/fnins.2023.1237920

**Published:** 2023-07-31

**Authors:** Thanh G. Phan, Rebecca Lim, Mirja Krause, Siow T. Chan, Hannah McDonald, Poh-Yi Gan, Shenpeng R. Zhang, Liz J. Barreto Arce, Jason Vuong, Tharani Thirugnanachandran, Benjamin Clissold, John Ly, Shaloo Singhal, Marie Veronic Hervet, Hyun Ah Kim, Grant R. Drummond, Euan M. Wallace, Henry Ma, Christopher G. Sobey

**Affiliations:** ^1^Clinical Trials, Imaging and Informatics Division, Stroke and Ageing Research, Department of Medicine, School of Clinical Sciences at Monash Health, Monash University, Clayton, VIC, Australia; ^2^Department of Neurology, Monash Health, Clayton, VIC, Australia; ^3^The Ritchie Centre, Hudson Institute of Medical Research, Clayton, VIC, Australia; ^4^Department of Obstetrics and Gynaecology, Monash University, Clayton, VIC, Australia; ^5^Department of Medicine, Centre for Inflammatory Diseases, Monash Medical Centre, School of Clinical Sciences, Monash University, Clayton, VIC, Australia; ^6^Department of Immunology, Monash Health, Monash Medical Centre, Clayton, VIC, Australia; ^7^Department of Microbiology, Anatomy, Physiology and Pharmacology, Centre for Cardiovascular Biology and Disease Research, School of Agriculture, Biomedicine and Environment, La Trobe University, Bundoora, VIC, Australia; ^8^Victorian Department of Health, Melbourne, VIC, Australia

**Keywords:** stem cell, clinical trial, ischemic stroke, phase I, allogeneic

In the published article, there was an error in the author list, and author “Mirja Krause” was erroneously excluded. The corrected author list and author contributions section are appears below.

“Thanh G. Phan^1, 2*^, Rebecca Lim^3, 4^, Mirja Krause^3, 4^, Siow T. Chan^3, 4^, Hannah McDonald^3, 4^, Poh-Yi Gan^5, 6^, Shenpeng R. Zhang^7^, Liz J. Barreto Arce^7^, Jason Vuong^1, 2^, Tharani Thirugnanachandran^1, 2^, Benjamin Clissold^1, 2^, John Ly^1, 2^, Shaloo Singhal^1, 2^, Marie Veronic Hervet^1, 2^, Hyun Ah Kim^7^, Grant R. Drummond^7^, Euan M. Wallace^4, 8^, Henry Ma^1, 2^ and Christopher G. Sobey^7^^*^”

## Author contributions

TP, HMa, RL, EW, and CS designed the trial protocol and were major contributors in the writing of the manuscript. JV performed image analysis. TT, BC, JL, and SS recruited patients and performed clinical assessments. RL, MK, SC, and HMc prepared cells for administration to patients. MH administered cell infusions and collected the patient data. P-YG, SZ, LB, and HK performed the analyses of blood samples for inflammatory markers. CS and GD analyzed the data. All authors contributed to the article and approved the submitted version.

The authors apologize for this error and state that this does not change the scientific conclusions of the article in any way. The original article has been updated.

